# CT Perfusion with Acetazolamide Challenge in C6 Gliomas and Angiogenesis

**DOI:** 10.1371/journal.pone.0121631

**Published:** 2015-03-17

**Authors:** Na Lu, Yue Di, Xiao-Yuan Feng, Jin-Wei Qiang, Jia-wen Zhang, Yong-gang Wang, Ying Liu

**Affiliations:** 1 Department of Radiology, Jinshan Hospital, Fudan University, Shanghai, China; 2 Department of Ophthalmology, Jinshan Hospital, Fudan University, Shanghai, China; 3 Department of Radiology, Huashan Hospital, Fudan University, Shanghai, China; 4 Department of Oncology, The Sixth People's Hospital, Shanghai Jiao Tong University, Shanghai, China; 5 Department of Pathology, Shanghai Medical College, Fudan University, Shanghai, China; Glasgow University, UNITED KINGDOM

## Abstract

**Background:**

This study was performed to investigate the correlation between CT perfusion with acetazolamide challenge and angiogenesis in C6 gliomas.

**Methods:**

Thirty-two male Sprague-Dawley rats were evaluated. The rats were divided randomly to four groups: eight rats with orthotopically implanted C6 gliomas at 10-days old (Group A), eight rats with gliomas at 14-days old (Group B), eight rats with gliomas at 18-days old (Group C), eight rats with orthotopically injected normal saline served as controls. CT perfusion was performed before and after administration of acetazolamide. Changes in perfusion parameters due to acetazolamide administration were calculated and analyzed.

**Results:**

Elevated carbon dioxide partial pressure and decreased pH were found in all 32 rats post acetazolamide challenge (*P*<0.01). Cerebral blood flow_pre-challenge_ was increased in group C (95.0±2.5 ml/100g/min), as compared to group B (80.1±11.3 ml/100g/min) and group A (63.1±2.1 ml/100g/min). Cerebral blood flow percentage changes were detected with a reduction in group C (54.2±4.8%) as compared to controls (111.3±22.2%). Cerebral blood volume _pre-challenge_ was increased in group C (50.8±1.7ml/100g), as compared to group B (45.7±1.9 ml/100g) and group A (38.2±0.8 ml/100g). Cerebral blood volume percentage changes were decreased in group C (23.5±4.6%) as compared to controls (113.5±30.4%). Angiogenesis ratio = [(CD105-MVD) / (FVIII-MVD)] ×100%. Positive correlations were observed between CD105-microvessel density, angiogenesis ratio, vascular endothelial growth factor, proliferation marker and cerebral blood flow_pre-challenge_, cerebral blood volume _pre-challenge_. Negative correlations were observed between CD105-microvessel density and cerebral blood flow percentage changes (*P*<0.01, correlation coefficient r=-0.788), cerebral blood volume percentage changes (*P*<0.01, r=-0.703). Negative correlations were observed between angiogenesis ratio, vascular endothelial growth factor, proliferation marker and cerebral blood flow percentage changes, cerebral blood volume percentage changes.

**Conclusion:**

Our findings suggest that CT perfusion with challenge can provide new insight into non-invasive assessment of rat C6 glioma angiogenesis.

## Introduction

Gliomas are common primary brain tumors[1]. The growth and metastasis of gliomas require adequate neovascularization[2]. The outcome for most patients with malignant gliomas is poor[3]. Therapies targeting neovascularization in malignant gliomas show promise in improving outcome[4, 5]. Microvessel density (MVD) has been used to predict treatment outcome[6, 7]. It has been reported that increased CD105-MVD correlates with shorter survival[8]. Angiogenesis ratio (AR) = [(CD105-MVD) / (FVIII-MVD)] ×100%. AR is established to determine the proliferating fraction of endothelial cells[9]. Thus, AR can be used as a predictive factor for invasion in gliomas. The up regulation of vascular endothelial growth factor (VEGF) plays an essential role during glioma angiogenesis[10]. Proliferation marker (Ki-67) is a nuclear antigen which appears to be one of the most rooted and widely used markers to estimate glioma cellular proliferation[11]. However, histological evaluation of glioma angiogenesis has limitations, such as sampling error and inter-observer variation[12]. Thus non-invasive assessment of angiogenesis is therefore a key element in the evaluation of gliomas[13]. Hence, research is urgent to develop imaging methods affording a comprehensive evaluation of glioma angiogenesis.

CT perfusion could provide valuable information regarding glioma vascularization[14]. Cerebral vascular reactivity (CVR) is expressed as cerebral blood flow (CBF) percentage changes from baseline under a vasodilator[15]. If the CBF percentage changes are decreased, pre-existing cerebral impairment is inferred. Acetazolamide is used as a vasodilator. It inhibits the enzyme carbonic anhydrase which is responsible for catalyzing the reversible reaction involving the hydration of carbon dioxide and the dehydration of carbonic acid[16]. Thus, CT perfusion with acetazolamide challenge has the potential to detect early CVR changes. It has been reported in our primary results that PCT with ACZ challenge was an ideal technique for quantitative evaluation of microcirculation in rat C6 glioma[17]. Therefore, this manuscript describes our initial experience on CT perfusion with acetazolamide to investigate correlations between CT perfusion parameters percentage changes and CD105-MVD, VEGF, Ki-67 values.

## Materials and Methods

### Ethics

Official guidelines for animal care were followed. Approval was obtained from the Huashan institutional review board of Fudan University (KY2011–001). Animals were housed and handled in accordance with the guide for the care and use of laboratory animals. Rats were anesthetized with intra-peritoneal 10% chloral hydrate (1 ml/300g). At the end of the experiment, the animals were sacrificed by means of injection of 10% formalin and physiological saline through left ventricle under anesthesia.

### Animal Preparation

The C6 glioma cell line (Institute of Biochemistry and Cell Biology, Chinese Academy of Sciences) kindly donated by J. M. Luo (Shanghai Cancer Research Institute), was cultured under standard conditions. From January 2010 to May 2014, CT perfusion with acetazolamide challenge was performed on 32 male Sprague-Dawley rats (age three months, weight 250–300g, selected randomly from Shanghai Laboratory Animal Center, Chinese Academy of Sciences, China). The rats were divided randomly to four groups: eight rats with orthotopically implanted C6 gliomas at 10-days old (Group A), eight rats with gliomas at 14-days old (Group B), eight rats with gliomas at 18-days old (Group C), eight rats with orthotopically injected normal saline at 14-days served as controls.

Prior to inoculation, the rats were anesthetized with intra-peritoneal chloral hydrate (40 mg/kg). Rats were securely placed onto a stereotactic head holder (KOPF 900, USA). A scalp incision was performed along the median line. A 1 mm diameter burr hole which was three millimeters right laterally and 1 millimeter frontal to bregma was drilled in the skull. In glioma group, a 10 μL cell suspension (1×10^6^ C6 glioma cells in Dulbecco’s modified Eagle’s medium complemented with 10% of calf serum) was injected into the right caudate nucleus at a depth of 5 mm under the skull to generate C6 glioma models, using a microsyringe. The 10 μL cell suspension was injected over a 10-minute period. The injection microsyringe was left in place for an additional 5 minutes to minimize spread of the solution along the injection track. In controls, a 10 μL normal saline instead of C6 glioma cells was injected into the right caudate nucleus over a 10-minute period. Five minutes after injection, the microsyringe was slowly removed in 5–10 minutes. The burr hole was plugged with bone wax (B/Braun, Aseculap AG, Germany) for mechanical control of hemorrhages in bone injuries. The tumor was then allowed to grow for 10-days (Group A), 14-days (Group B), 18-days (Group C). A total of 24 tumor-bearing rats and eight rats in controls were performed CT perfusion imaging.

### CT perfusion imaging with challenge Procedure

All examinations were performed with a 256-slice scanner (Brilliance iCT, Philips Medical Systems, Cleveland, OH, USA). The rats were anesthetized with intra-peritoneal chloral hydrate (40 mg/kg). An intravenous catheter was used to cannulate the vena dorsalis penis of rats for contrast medium administration. The baseline CT acquisition provided wide coverage to include the glioma. This first scan basically served as a localizer to make sure that the whole rat brains would be covered by the perfusion imaging field. Then CT perfusion imaging was performed in a continuous scan pattern, 512×512 pixels. Using a high pressure syringe, 2 ml of Omnipaque 300 (GE Healthcare, Shanghai, China) was administered at a flow rate (1ml· sec^−1^) in the vena dorsalis penis. The CT perfusion parameters were as follows. Slice thickness was two millimeter. Voltage was 80 kVp. Current was 50 mAs. Pitch was 0.9 mm. Rotation time was 0.5 s. The protocol for CT perfusion imaging contained of a total of 49 acquisitions, which were acquired over a time period of 210 seconds. An acquisition was performed every two seconds for the first 45 acquisitions. An acquisition was performed every 30 seconds for the following four acquisitions. Carbon dioxide partial pressure (PaCO_2_) and pH pre and post acetazolamide challenges were recorded.

The protocol for CT perfusion imaging with challenge contained four steps. Firstly, rats underwent brain CT perfusion imaging pre-challenge. Secondly, rats waited one hour to minimize the effects of Omnipaque. Thirdly, rats were injected acetazolamide (0.3 mg/300g) intravenously. Finally, rats underwent brain CT perfusion imaging post-challenge 15 minutes after intravenous acetazolamide.

### CT perfusion imaging with Challenge Analysis

The raw data were processed using CT brain perfusion software, which could provide time density curves and color maps. The arterial input was set on the middle cerebral artery. According to deconvolution algorithm, color maps of different CT perfusion parameters with rainbow color ramps were generated.

Regions of interest (ROIs) were positioned on the gliomas, avoiding small vessels and necrosis. Three ROIs were chosen, and the mean values of perfusion parameters were recorded to minimize the effect of heterogeneity. CBF and cerebral blood volume (CBV) were recorded pre and post acetazolamide challenge. From these results, CBF percentage changes and CBV percentage changes were calculated.

### Histologic Analysis

The rats were anesthetized with intra-peritoneal chloral hydrate (40 mg/kg). At the end of the experiment, the animals were sacrificed by means of injection of 10% formalin and physiological saline through left ventricle under anesthesia. Their brains were resected, fixed in 10% formalin, and embedded in paraffin in two blocks, which were cut at the level of the injection site. A coronal 5 μm thick slice of brain tissue from each rat was cut and stained according to the standard hematoxylin eosin staining protocol.

Specimens were stained for CD105 (1: 30), VEGF (1: 200), Ki-67 (1: 100), and endothelial cell marker FVIII (1: 200) by immunohistochemistry. MVD was defined as the mean value of the vessel count in five separate fields (200-fold magnification)[18]. Then, CD105-MVD, VEGF, Ki-67 and FVIII-MVD in gliomas were recorded.

### Statistical Analysis

Percentage changes were calculated as follows: percentage changes = [(parameters_post-challenge_-parameters_pre-challenge_)/parameters_pre-challenge_]×100%,where parameters_pre-challenge_ and parameters_post-challenge_ represented the parameters before and after acetazolamide challenge. AR = [(CD105-MVD) / (FVIII-MVD)] ×100%.

The basic data were expressed as mean ± standard deviations. The statistical analysis was performed using the SPSS 17.0 package (SPSS Inc., Chicago, IL, USA). Paired-samples *t* tests were used to compare perfusion parameters pre and post acetazolamide challenge in C6 gliomas. ANOVA tests were used to compare the parameters among different groups, after confirmation of normal distribution of the data. In the multiple comparison procedures, a *P*-value less than 0.01 was considered significant after Bonferroni correction. Pearson correlation coefficients were used to investigate relationships between CBF percentage changes, CBV percentage changes and AR, CD105-MVD, VEGF, Ki-67. A *P*-value less than 0.05 was considered significant.

## Results

### PaCO_2_ and pH pre and post acetazolamide challenge

CT perfusion was technically feasible in 32 cases of this study. Elevated PaCO_2_ and decreased pH were found in all 32 rats post acetazolamide challenge (*P*<0.01, Table 1). Twenty-four C6 rats were histopathologically proven to have gliomas.

**Table 1:** PaCO_2_ and pH pre and post acetazolamide (n = 32).

	pH	PaCO_2_(Kpa)
Pre challenge	7.3±0.2	4.7±0.2
Post challenge	6.7±0.2	7.9±0.2
*t*	11.7	-56.0
*P*	0.000	0.000

### CT perfusion imaging parameters pre and post acetazolamide challenge

**Fig 1:**
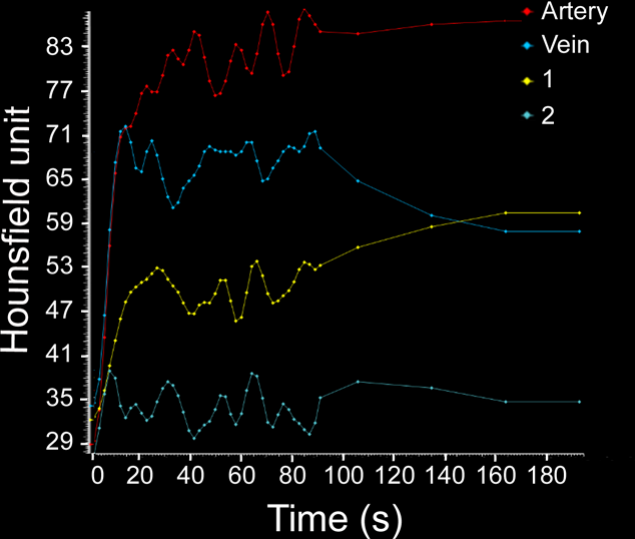
CT perfusion time density curves in a rat with C6 gliomas (group B) pre challenge. ROI of the red artery curve was placed on the right middle cerebral artery. ROI of the blue vein curve was placed on the superior sagittal sinus. ROI of the yellow curve (curve one) was placed on C6 glioma. ROI of the green curve (curve two) was placed on contralateral normal brain tissue. Time density curves showed the peak of the yellow curve was higher than that of the green curve.

**Fig 2:**
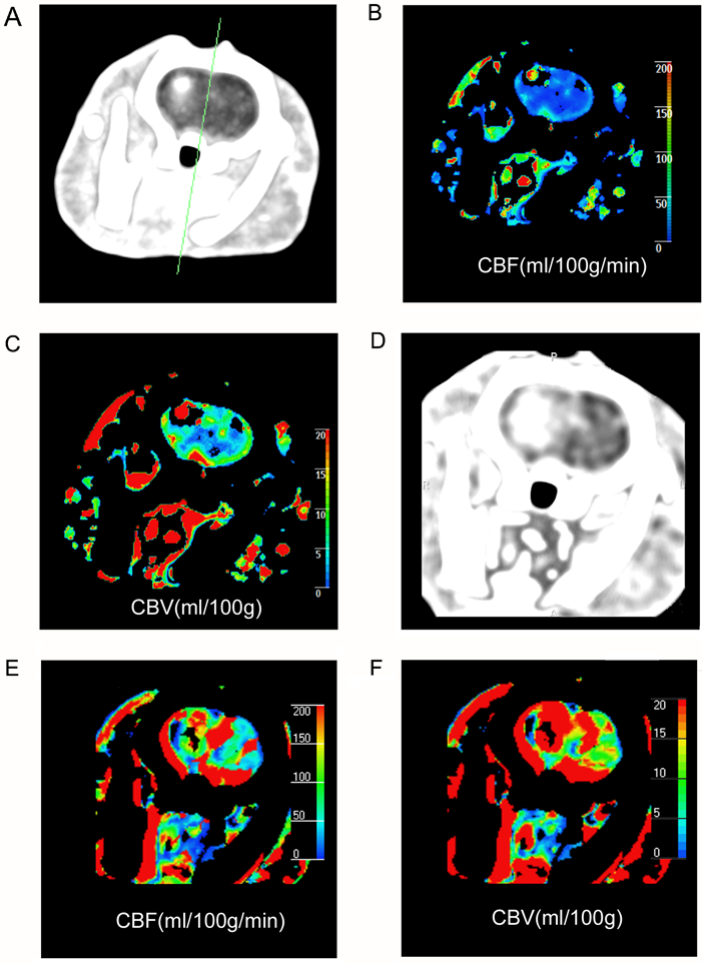
CT perfusion color maps in a rat with C6 gliomas (group B) pre and post acetazolamide challenge. (A-C) Maximun intensity projection image, perfusion maps for cerebral blood flow, cerebral blood volume of C6 glioma pre acetazolamide stimuli. (D-F) Maximun intensity projection image, perfusion maps for cerebral blood flow, cerebral blood volume of C6 glioma post acetazolamide challenge. Glioma was obviously enhanced in maximum intensity projection map on right caudate area (A). Higher CBF of glioma on right caudate was shown on CBF map than contralateral normal tissue (B). Higher CBV of glioma on right caudate was shown on CBV map than contralateral normal tissue (C). CT perfusion maps post challenge showed higher density, CBF and CBV than pre challenge (D-F).

In gliomas, CBF_pre-challenge_ was increased in group C (95.0±2.5 ml/100g/min), as compared to group B (80.1±11.3 ml/100g/min) and group A (63.1±2.1 ml/100g/min). CBV_pre-challenge_ was increased in group C (50.8±1.7 ml/100g), as compared to group B (45.7±1.9 ml/100g) and group A (38.2±0.8 ml/100g) in gliomas. CBF and CBV values were higher post acetazolamide challenge than pre challenge in all groups, as shown in Table 2. Time density curves of a rat in group B before challenge were shown on Fig. 1. CT perfusion maps of a rat in group B before and after challenge were shown on Fig. 2.

**Table 2:** CT perfusion parameters pre and post acetazolamide challenge in C6 gliomas (Mean ± SD).

	n	CBF_pre-challenge_ (ml/100g/min)	CBF_post-challenge_ (ml/100g/min)	CBV_pre-challenge_ (ml/100g)	CBV_post-challenge_ (ml/100g)
Controls	8	54.8±3.9	118.7±14.4^##^	8.6±0.6	19.2±1.2^##^
Group A	8	63.1±2.1	162. 9±2.7^##^	38.2±0.8	56.5±2.1^##^
Group B	8	80.1±11.3* ^Δ^	154.4±7.5^##^	45. 7±1.9* ^Δ^	58.2±6.7^##^
Group C	8	95.0±2.5* ^Δ^ ^#^	146.4±2.3^##^	50.8±1.7* ^Δ^ ^#^	62.7±1.9^##^

*P*<0.01 versus the group pre acetazolamide challenge (^#^)

*P*<0.01 versus controls (*)

*P*<0.01 versus Group A (^Δ^)

*P*<0.01 versus Group B (^#^)

### CT perfusion imaging with challenge parameters

CBF percentage changes were detected with a reduction in group C (54.2±4.8%) as compared to controls (111.3±22.2%). CBV percentage changes were detected with a reduction in group C (23.5±4.6%) as compared to controls (113.5±30.4%). CBV percentage changes in group A and B were lower than controls. These results were shown in Table 3.

**Table 3:** CBF percentage changes and CBV percentage changes in C6 gliomas (Mean ± SD).

	N	CBF percentage changes(%)	CBV percentage changes(%)
Controls	8	111.3±22.2	113.5±30.4
Group A	8	128.5±9.2	47.9±7.3*
Group B	8	96.3±30.4	27.3±12.7*
Group C	8	54.2±4.8* ^Δ^ ^#^	23.5±4.6*

*P*<0.01 versus controls (*)

*P*<0.01 versus Group A (^Δ^)

*P*<0.01 versus Group B (^#^)

### Angiogenesis in C6 gliomas

Group B and C had higher CD105-MVD, VEGF, Ki67 than group A (*P*<0.01). Group C had higher CD105-MVD, VEGF than group B (*P*<0.01). Histopathological values in group A, group B and group C were shown in Table 4.

**Table 4:** Histopathological values CD105-MVD, AR, VEGF and Ki67 in group A, group B and group C of C6 gliomas (Mean ± SD).

	n	CD105-MVD(%)	AR(%)	VEGF(%)	Ki67(%)
Group A	8	1.3±0.5	0.4±0.2	3.8±0.4	7.6±2.3
Group B	8	6.4±2.3*	0.5±0.1	7.9±2.8*	16.5±5.7*
Group C	8	17.9±1.3* ^#^	0.6±0.1	12.6±1.0* ^#^	20.5±2.5*

*P*<0.01 versus Group A (*)

*P*<0.01 versus Group B (^#^)

### Correlations between CT perfusion with challenge parameters and angiogenesis

**Fig 3:**
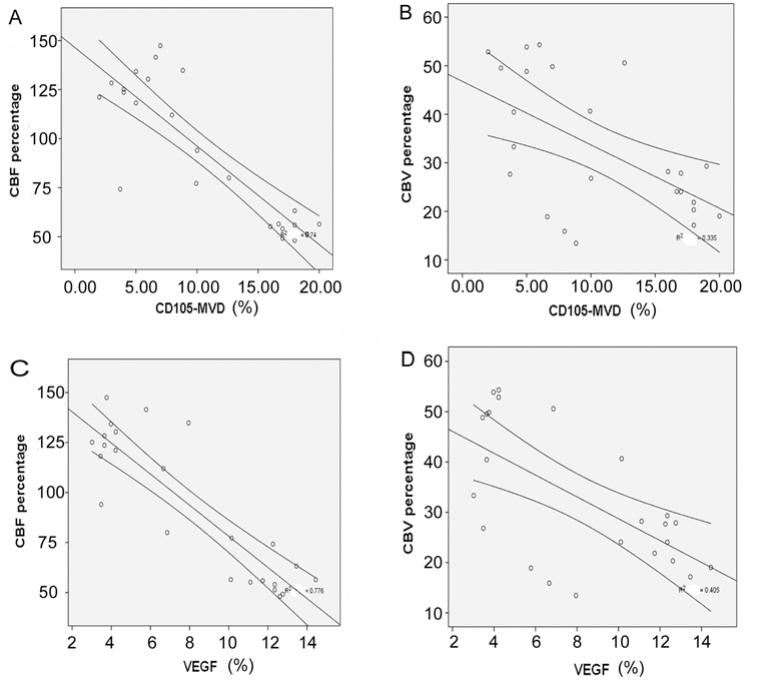
Correlation diagrams between CD105-MVD, VEGF and CBF percentage changes, CBV percentage changes. Negative correlation was shown in C6 gliomas between CBF percentage changes and CD105-MVD (A). Negative correlation was shown in C6 gliomas between CBV percentage changes and CD105-MVD (B). Negative correlation was shown between CBF percentage changes and VEGF (C). Negative correlation was shown in C6 gliomas between CBV percentage changes and VEGF (D). The middle lines on each graph showed the mean value of correlation. The upper and lower lines which were on both sides of the middle lines on each graph showed the 95% confidence interval of the correlation.

**Fig 4:**
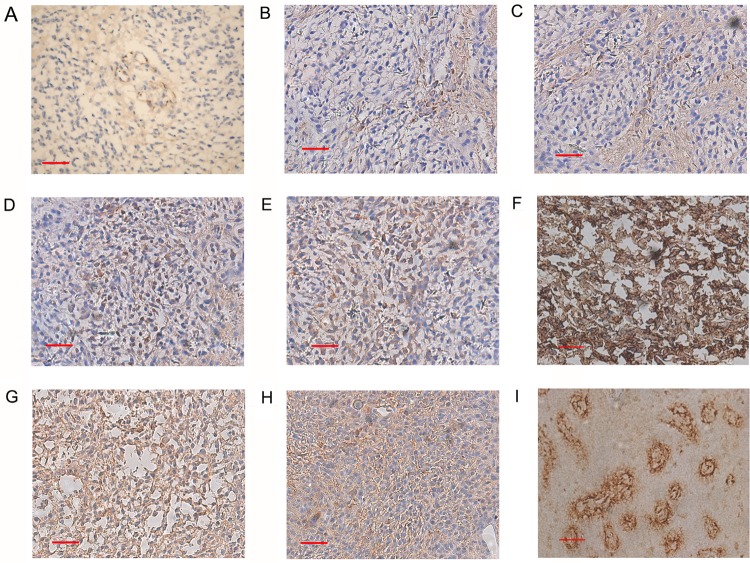
Histologic images of group A, B and C. CD105-MVD in rat C6 glioma group A(A), group B(B) and group C (C). VEGF in rat C6 glioma group A(D), group B(E) and group C (F). FVIII-MVD in rat C6 glioma group A(G), group B(H) and group C (I). Scale bar = 50um.

Positive correlations were found between CD105-MVD, AR, VEGF, Ki-67 and CBF_pre-challenge_, CBV_pre-challenge_. Negative correlations were observed between CD105-MVD and CBF percentage changes (*P*<0.01, correlation coefficient r = -0.788) and CBV percentage changes (*P*<0.01, r = -0.703). Negative correlations were observed between AR, VEGF, Ki-67 and CBF percentage changes, CBV percentage changes, as shown in Table 5 (*P*<0.01). The negative correlation diagrams between CD105-MVD, VEGF and CBF percentage changes, CBV percentage changes were demonstrated in Fig. 3. Histologic images of group A, B and C were shown on Fig. 4. Our data were made available in supporting information files S1– S3 Datasets.

**Table 5:** Pearson Correlation of PCT with challenge parameters and histopathological values in C6 gliomas.

	r(CBF percentage changes)	r(CBV percentage changes)	r(CBF_pre challenge_)	r(CBV_pre challenge_)
CD105-MVD	-0.788**	-0.703**	0.819**	0.869**
AR	-0.434*	-0.532**	0.461*	0.479*
VEGF	-0.881**	-0.636**	0.871**	0.910**
Ki67	-0.842**	-0.546**	0.858**	0.820**

* Correlation was significant at the 0.05 level.

**Correlation was significant at the 0.01 level.

## Discussion

### CT perfusion with acetazolamide challenge

CT perfusion is performed on the basis of the central volume principle by monitoring the first pass of a bolus of iodinated contrast material[19, 20]. CT perfusion is a valuable technique that has been successfully applied to the clinical management of patients with ischemic stroke[21]. In gliomas, CT perfusion has a role in accessing hemodynamics and mapping of glioma microcirculation[22]. Acetazolamide, a potent inhibitor of the reversible hydration of CO_2_ catalyzed by the enzyme carbonic anhydrase, is a cerebral stimulus[23]. Acetazolamide challenge is simple and safe[24]. Nowadays, acetazolamide is used to increase CBF in single-photon emission computed tomography (SPECT)[25].

Elevated PaCO_2_ and decreased pH post acetazolamide challenge were found in this study. This proved acetazolamide acted as a stimulus to increase CBF in CT perfusion imaging. CT perfusion with acetazolamide challenge was promising for the evaluation of cerebral hemodynamics in patients with cerebrovascular steno-occlusive disease[26, 27]. The availability and rapidity of CT perfusion with acetazolamide challenge could result in potential non-invasive assessment of human gliomas. MR perfusion imaging and SPECT were also exploited for cerebral perfusion. It was reported that elevated perfusion parameters by MR perfusion correlated to tumor angiogenesis[28]. This was coincident with our study, which proved that elevated CBF_pre-challenge_ and CBV_pre-challenge_ by CT perfusion with challenge correlated to glioma angiogenesis. However, the relationship between the gadolinium concentration and the measured signal in MR perfusion was logarithmic, and therefore, only relative perfusion measurements could be gotten by MR perfusion imaging[29]. Besides, MR perfusion imaging were affected by hemorrhage, calcification and post-surgical metallic implants[22]. CT perfusion showed linear relationship between attenuation changes on CT and tissue concentration of contrast medium and quantitative results[30]. SPECT combined with CT or MRI could be beneficial[31]. It was reported that there was a high correlation between SPECT and CT perfusion[32]. However, SPECT might be unavailable outside large centers. SPECT also had low resolution. Therefore, we used CT perfusion with acetazolamide challenge in the present study.

### CT perfusion with challenge and angiogenesis

Post acetazolamide challenge, higher CBF and CBV were found than pre challenge. This proved the acetazolamide challenge was effective as reported[17]. Our results were that the CBF percentage changes and CBV percentage changes of 10-day group (A) followed by the 14-day group (B), and finally by the 18-day group (C). These data implied that CVR of 10-day group (A) followed by the 14-day group (B), and finally by the 18-day group (C). It was reported that CVR mapping could effectively demonstrate areas of neurovascular uncoupling[28]. We found CBF percentage changes and CBV percentage changes were associated with tumor angiogenesis. This proved that CBF percentage changes and CBV percentage changes might associate with immature vessels percentage in C6 gliomas. This was coincident with Vagal AS et al.’s investigation which proved that hemodynamic changes could be inferred by abnormal CBF percentage changes and CBV percentage changes[33]. In our study, Group C had higher VEGF than group B (*P*<0.01). Group B and C had higher VEGF, Ki67 than group A (*P*<0.01). Our results showed negative correlations between CBF percentage changes, CBV percentage changes and AR, CD105-MVD, VEGF, Ki-67. This implied that CVR in C6 glioma was related to angiogenesis. In controls, CBF percentage changes and CBV percentage changes were high. This implied there was normal cerebral autoregulation in controls with orthotopically saline injected. Thus the decrease of CVR might be because of angiogenesis, not the response of surgery. Positive correlations were found between CBF_pre-challenge_, CBV_pre-challenge_ and glioma angiogenesis. Consequently, we deduced that CT perfusion imaging with acetazolamide challenge was related to glioma angiogenesis. C6 gliomas did not have a robust increase in CBF and CBV after acetazolamide challenge. The reason might be C6 glioma vessels did not have the full ability to dilate. There was vascular dysfunction in gliomas. Thus, cerebral autoregulation impairment was associated with glioma angiogenesis. Chen A et al.’s reported that CBF percentage changes were used to evaluate hemodynamic impairment[16]. That was coincident with our study. In this study, mean transit time percentage changes, time to peak percentage changes and permeability percentage changes were not used, as the three parameters did not respond well in gliomas as reported [17]. However, this was not coincident with Smith LM et al.’s investigation[34]. The reason might be that the results were affected by sampling error. So perfusion parameters percentage changes such as mean transit time percentage changes and permeability percentage changes could be further investigated. We only investigated ROIs on gliomas without the edema adjacent to the gliomas in this study. This could be further investigated. Small blood vessels of tumor periphery and necrosis of tumor central area might affect CT perfusion parameters[17]. Besides, gliomas were quite heterogeneous. Hence, three ROIs were chosen in this study and the mean values of perfusion parameters were used. The reason why animal model groups were established at 10-days old, 14-days old and 18-days old was that C6 gliomas had enough angiogenesis and changes to be detected between groups at 10-days old, 14-days old and 18-days old. Andaluz et al. deduced that CT perfusion with acetazolamide challenge might have a key role in the evaluation of cerebral perfusion and CVR in adult Moyamoya patients[35]. Fulesdi et al. assessed CVR using acetazolamide challenge on patients with severe sepsis[36]. This made it feasible to use CT perfusion imaging with acetazolamide challenge on patients. Thus, the next reasonable hypothesis would be CT perfusion with acetazolamide challenge might have a role in the evaluation of angiogenesis in patients with gliomas. Jain et al. deduced that CT perfusion correlated with VEGF, which was coincident with our results[37].

Our results were promising to provide useful information for glioma angiogenesis. Nevertheless, there were a few issues regarding the CT perfusion with acetazolamide challenge. Firstly, we found CBF percentage changes and CBV percentage changes were associated with tumor angiogenesis in rat C6 gliomas. However, there were a host of differences between human and rat. Thus the investigation of CT perfusion imaging with acetazolamide on patients with gliomas was urgent. Secondly, the radiation dose linearly increased with the number of acquisitions. Although voltage and current were lowered in our study, CT perfusion imaging with acetazolamide should not be used on children.

## Conclusion

In conclusion, our findings showed an inverse correlation between CBF percentage changes, CBV percentage changes and angiogenesis. Additionally, both CBF_pre-challenge_ and CBV_pre-challenge_ showed a positive correlation with C6 glioma angiogenesis. These findings proved that CT perfusion imaging with challenge could provide new insight into non-invasive assessment of tumor angiogenesis in rat C6 glioma.

## Supporting Information

S1 Dataset This is the dataset for table 1. (DOC)

S2 Dataset This is the dataset for table 2 and 3. (DOC)

S3 Dataset This is the dataset for table 4 and 5. (DOC)
