# Supplementary material for: CT Perfusion with Acetazolamide Challenge in C6 Gliomas and Angiogenesis
Source: PLoS One. 2015 Mar 17;10(3):e0121631. doi: 10.1371/journal.pone.0121631 (PMC4363697; doi:10.1371/journal.pone.0121631)
Supplement: S1 Dataset — (DOC) [file pone.0121631.s001.doc]

| Number | Mouse | Location | Days | Pathology | PHpreacetazolamide | PCO_2_preacetazolamide | PHpostacetazolamide | PCO_2_postacetazolamide |
| --- | --- | --- | --- | --- | --- | --- | --- | --- |
| 1 | 7 | 0.00 | 1 | 1 | 7.13 | 4.51 | 6.84 | 7.92 |
| 2 | 17 | 0.00 | 1 | 1 | 7.43 | 4.62 | 6.93 | 7.94 |
| 3 | 18 | 0.00 | 1 | 1 | 7.12 | 4.69 | 6.48 | 7.93 |
| 4 | 19 | 0.00 | 1 | 1 | 7.45 | 4.68 | 6.74 | 7.89 |
| 5 | 45 | 0.00 | 1 | 1 | 7.83 | 4.89 | 6.95 | 7.92 |
| 6 | 44 | 0.00 | 1 | 1 | 7.15 | 4.72 | 6.84 | 8.02 |
| 7 | 36 | 0.00 | 1 | 1 | 7.53 | 4.62 | 6.85 | 8.00 |
| 8 | 37 | 0.00 | 1 | 1 | 7.93 | 4.64 | 6.93 | 8.04 |
| 9 | 15 | 2.00 | 2 | 2 | 7.10 | 4.68 | 6.38 | 7.98 |
| 10 | 20 | 2.00 | 2 | 2 | 7.03 | 4.67 | 6.83 | 7.49 |
| 11 | 21 | 2.00 | 2 | 2 | 7.42 | 4.92 | 6.75 | 7.78 |
| 12 | 23 | 2.00 | 2 | 2 | 7.92 | 4.46 | 6.92 | 8.42 |
| 13 | 24 | 2.00 | 2 | 2 | 7.11 | 4.62 | 6.27 | 7.68 |
| 14 | 25 | 2.00 | 2 | 2 | 7.24 | 4.47 | 6.35 | 7.92 |
| 15 | 26 | 2.00 | 2 | 2 | 7.25 | 4.48 | 6.27 | 7.49 |
| 16 | 27 | 2.00 | 2 | 2 | 7.14 | 4.46 | 6.28 | 8.24 |
| 17 | 16 | 2.00 | 3 | 2 | 7.15 | 4.72 | 6.92 | 7.94 |
| 18 | 10 | 2.00 | 3 | 2 | 7.34 | 4.72 | 6.83 | 8.22 |
| 19 | 11 | 2.00 | 3 | 2 | 7.14 | 4.66 | 6.55 | 7.89 |
| 20 | 9 | 2.00 | 3 | 2 | 7.24 | 4.62 | 6.83 | 7.98 |
| 21 | 8 | 2.00 | 3 | 2 | 7.29 | 4.92 | 6.93 | 7.73 |
| 22 | 13 | 2.00 | 3 | 2 | 7.28 | 4.82 | 6.83 | 7.82 |
| 23 | 12 | 2.00 | 3 | 2 | 7.03 | 4.62 | 6.94 | 7.70 |
| 24 | 14 | 2.00 | 3 | 2 | 7.04 | 4.62 | 6.67 | 7.59 |
| 25 | 28 | 2.00 | 4 | 2 | 7.00 | 4.78 | 6.82 | 7.98 |
| 26 | 29 | 2.00 | 4 | 2 | 7.08 | 4.92 | 6.82 | 7.34 |
| 27 | 30 | 2.00 | 4 | 2 | 7.25 | 4.29 | 6.82 | 8.03 |
| 28 | 31 | 2.00 | 4 | 2 | 7.24 | 4.82 | 6.72 | 7.80 |
| 29 | 32 | 2.00 | 4 | 2 | 7.22 | 4.67 | 6.92 | 7.57 |
| 30 | 33 | 2.00 | 4 | 2 | 7.00 | 4.83 | 6.62 | 7.79 |
| 31 | 34 | 2.00 | 4 | 2 | 7.13 | 4.82 | 6.62 | 7.93 |
| 32 | 35 | 2.00 | 4 | 2 | 7.22 | 4.27 | 6.82 | 8.03 |
