# Supplementary material for: CT Perfusion with Acetazolamide Challenge in C6 Gliomas and Angiogenesis
Source: PLoS One. 2015 Mar 17;10(3):e0121631. doi: 10.1371/journal.pone.0121631 (PMC4363697; doi:10.1371/journal.pone.0121631)
Supplement: S3 Dataset — (DOC) [file pone.0121631.s003.doc]

| Num  ber | Mou  se | Days | Patho | BVpreac  etazolamide | BVpostac  etazolamide | BFpreaceta  zolamide | BFpostaceta  zolamide | BVpercentag  echanges | BFpercent  agechanges | CD105 | AR | VEGF | Ki67 | SMA | FVIII |
| --- | --- | --- | --- | --- | --- | --- | --- | --- | --- | --- | --- | --- | --- | --- | --- |
| 1 | 15 | 2 | 2 | 37.85 | 56.33 | 65.79 | 163.53 | 48.82 | 118.16 | 1.00 | 0.33 | 3.45 | 10.00 | 1.00 | 3.00 |
| 2 | 20 | 2 | 2 | 38.42 | 57.45 | 62.39 | 162.46 | 49.53 | 128.34 | 1.00 | 0.50 | 3.65 | 9.00 | 2.00 | 2.00 |
| 3 | 21 | 2 | 2 | 39.53 | 55.52 | 66.34 | 168.35 | 40.45 | 123.62 | 1.00 | 0.50 | 3.65 | 11.00 | 1.00 | 2.00 |
| 4 | 23 | 2 | 2 | 37.62 | 57.89 | 60.82 | 162.45 | 53.88 | 134.22 | 1.00 | 0.25 | 3.98 | 8.00 | 1.40 | 4.00 |
| 5 | 24 | 2 | 2 | 38.18 | 58.92 | 62.48 | 163.89 | 54.32 | 130.30 | 1.00 | 0.20 | 4.23 | 7.00 | 1.20 | 5.00 |
| 6 | 25 | 2 | 2 | 37.24 | 56.93 | 63.43 | 160.24 | 52.87 | 121.09 | 1.00 | 0.50 | 4.22 | 5.00 | 1.30 | 2.00 |
| 7 | 26 | 2 | 2 | 37.93 | 56.83 | 60.41 | 159.47 | 49.83 | 147.42 | 2.00 | 0.18 | 3.76 | 6.00 | 1.40 | 11.00 |
| 8 | 27 | 2 | 2 | 39.07 | 52.09 | 63.41 | 162.76 | 33.32 | 125.13 | 2.00 | 0.67 | 3.02 | 5.00 | 1.70 | 3.00 |
| 9 | 16 | 3 | 2 | 46.26 | 65.07 | 87.60 | 155.20 | 40.66 | 77.16 | 4.94 | 0.58 | 10.15 | 17.67 | 1.57 | 8.50 |
| 10 | 10 | 3 | 2 | 45.23 | 56.12 | 96.64 | 151.23 | 24.08 | 56.49 | 4.68 | 0.51 | 10.11 | 21.89 | 1.89 | 9.25 |
| 11 | 11 | 3 | 2 | 46.71 | 70.34 | 84.07 | 151.32 | 50.59 | 80.00 | 4.61 | 0.50 | 6.86 | 18.12 | 1.28 | 9.31 |
| 12 | 9 | 3 | 2 | 47.40 | 60.51 | 82.18 | 143.19 | 27.66 | 74.23 | 3.71 | 0.27 | 12.26 | 22.08 | 1.79 | 13.59 |
| 13 | 8 | 3 | 2 | 46.98 | 54.45 | 78.73 | 166.90 | 15.90 | 111.99 | 7.95 | 0.50 | 6.66 | 10.17 | 1.93 | 15.97 |
| 14 | 13 | 3 | 2 | 44.17 | 50.11 | 64.16 | 150.66 | 13.45 | 134.82 | 8.84 | 0.57 | 7.95 | 5.92 | 1.94 | 15.46 |
| 15 | 12 | 3 | 2 | 46.71 | 55.54 | 63.54 | 153.44 | 18.90 | 141.49 | 6.61 | 0.45 | 5.78 | 19.69 | 1.50 | 14.77 |
| 16 | 14 | 3 | 2 | 41.87 | 53.10 | 84.12 | 163.19 | 26.82 | 94.00 | 10.01 | 0.61 | 3.49 | 16.37 | 1.40 | 16.51 |
| 17 | 28 | 4 | 2 | 53.76 | 62.98 | 90.53 | 147.74 | 17.15 | 63.19 | 18.00 | 0.72 | 13.46 | 19.00 | 1.30 | 25.00 |
| 18 | 29 | 4 | 2 | 50.32 | 64.35 | 96.24 | 143.53 | 27.88 | 49.14 | 17.00 | 0.55 | 12.76 | 18.00 | 1.20 | 31.00 |
| 19 | 30 | 4 | 2 | 50.78 | 65.10 | 95.27 | 147.83 | 28.20 | 55.17 | 16.00 | 0.67 | 11.11 | 17.00 | 1.20 | 24.00 |
| 20 | 31 | 4 | 2 | 49.67 | 59.12 | 96.24 | 150.53 | 19.03 | 56.41 | 20.00 | 0.57 | 14.45 | 22.00 | 1.00 | 35.00 |
| 21 | 32 | 4 | 2 | 48.21 | 62.34 | 95.63 | 144.75 | 29.31 | 51.36 | 19.00 | 0.53 | 12.35 | 23.00 | 1.70 | 36.00 |
| 22 | 33 | 4 | 2 | 52.34 | 62.98 | 98.53 | 145.89 | 20.33 | 48.07 | 18.00 | 0.44 | 12.62 | 21.00 | 1.80 | 41.00 |
| 23 | 34 | 4 | 2 | 51.32 | 63.67 | 95.35 | 146.86 | 24.06 | 54.02 | 17.00 | 0.53 | 12.35 | 24.00 | 1.30 | 32.00 |
| 24 | 35 | 4 | 2 | 50.34 | 61.34 | 92.54 | 144.32 | 21.85 | 55.95 | 18.00 | 0.49 | 11.74 | 20.00 | 1.60 | 37.00 |
